# Synergies between digital construction technologies in smart buildings and smart city development to meet building users’ expectations

**DOI:** 10.1016/j.heliyon.2024.e28585

**Published:** 2024-04-12

**Authors:** Mohammad Mayouf, Fiaz Afsar, Ayzah Iqbal, Vahid Javidroozi, Saeed Reza Mohandes

**Affiliations:** a Birmingham City University, Faculty of Computing, Engineering and the Built Environment, UK; b University of Manchester, Infrastructure and Resilience, Department of Mechanical, Aerospace & Civil Engineering, UK

**Keywords:** BIM, IoT, Blockchain, Smart buildings, Smart city, Building users

## Abstract

In smart buildings, digital construction technologies can support more efficient management of data and information related to building components. This paper aims to draw a robust linking mechanism between digital construction technologies that support smart buildings and smart city development to satisfy building users' expectations. Data was attained using a qualitative approach via secondary data from literature and primary data in the context of case study with building users. The study suggests the importance of recognising single/multi-purposed data to support better synergy between digital construction technologies and in smart buildings and smart city development to satisfy building users’ expectations.

## 1 Introduction

In today's society, one of the major demands of nowadays society is the use of technologies to manage a large amount of data so that it informs decision-making to meet citizens' demands [1]. In response to this, striving to make cities smart became a necessity so that demanding sectors such as housing, transportation and health can be optimised and improved. Therefore, since the early 2000s, the concept of a ‘smart city’ has been introduced [1,2]. The intended aims of a smart city is to provide accurate information in real-time to inform different sectors [3]. Within the built environment context, smart buildings [4] can be seen as one of the most important contributing parts to the requirement of Smart City Development (SCD). As a sub-set of smart environments, which forms a core sector in smart city development, smart buildings are supported by technological infrastructure that use data/information to improve the performance [5]. In recent years, many digital construction technologies [6] have supported enabling the infrastructure to prepare buildings in becoming smart, such as the Building Information Modelling (BIM) [7], Internet of Things (IoT) [8], and Blockchain technology [9]. These digital construction technologies can be recognised as key infrastructure components in smart buildings and enabling components in the context of smart cities.

Although vast research efforts have logically attempted to link smart city to many digital construction technologies such as BIM, IoT or even Blockchain [10], evidence of how this synergy provide value for building users in smart buildings is considerably limited. One of the underlying reasons is that data/information related to the built environment and data/information used for smart city development have been developed in different time-frames and by various stakeholders [7]. Another reason is the limited evidence of aligning outcomes from digital construction technologies including BIM, IoT and even blockchain in terms how they satisfy building users' needs and expectations within the context of smart buildings. Building users' expectations can be recognised as a core requirement at a building level, and also a smart city level. However, building users' expectations are not fixed, and in many cases, are complex to be holistically achieved [11], and building users' expectations can change from one building to another. Thus, despite the association between research available on digital construction technologies and smart cities, there is an importance to shed the light onto the synergy between them to satisfy building users' expectations. This will support rationalising a more informed implementation of digital construction technologies across construction projects, and more importantly, drawing more robust connections with SCD requirements. Thus, this research aims to draw a robust linking mechanism between digital construction technologies that support smart buildings and Smart City Development to satisfy building users’ expectations.

## 2 Research background

### 2.1 Digital construction technologies: BIM, IoT and blockchain

For nearly two decades, digitalisation within the construction industry across the whole life cycle has been on the rise. The main impact of digitalisation is providing an improved mechanism towards the management and automation and security of data/information. BIM can be recognised as one of the major digitalisation breakthroughs that provided solid path towards integrated project delivery. The concept of ‘BIM’ has gained momentum within the Architecture, Engineering, Construction and Operation (AECO) literature due to technological innovation in construction, but more so, due to an increasing understanding of its potential benefits. Despite no agreement amongst academics, it can be stated that BIM is an Information Technology (IT) orientated tool/process which provides a multi-accessible 3D digital modelling platform for built assets [12], essentially to share, exchange, store, and manage building information in an interoperable fashion [13]. The degree to which BIM is utilised by organisations and people, determines its definition in that given situation, thereby suggesting its definition is not stagnant [14]. [15] described BIM as a shift within the AECO industries due to its ability in enhancing collaboration and coordination between project stakeholders, inevitably changing modern ways of work and processes, for instance by improving communication and problem detection. In the context of smart buildings, many studies have pointed out the value of integrating BIM with IoT to support automated solutions [16]. explained the link between BIM and IoT integration is quite an innovative process, as there is a capability of the BIM software to integrate actual or future smart buildings in IoT. For instance, many studies (e.g. Refs. [17,18] have developed an integrated BIM-IoT solution to optimise temperature and humidity. Other studies (e.g. Ref. [19] provided IoT and BIM-based systems to support thermal comfort in smart buildings. Beyond smart buildings, BIM can help address several smart city challenges related to housing, climate change, traffic congestion, smart road maintenance, ageing infrastructure, etc.

Compared to BIM, where the primary value is an integrated management of data/information of a building, IoT is a system of multiple interconnected devices, digital and mechanical machines, animals, or people with unique identifiers [UIDs]. These connected things can transfer and share data over a network without demanding human-to-human or human-to-computer communication [20]. IoT can be seen as the connectivity between the digital and the physical world [21]. Other studies [[22], [23], [24]] defined IoT as a developing technology that offers a connected network that enables things to be connected at anyplace, anytime, with anyone, ideally by using any connection, any path, and any service. In this case, IoT enables subsystems to communicate with each other incessantly, creating large, connected systems capable of generating, delivering, collecting, analysing, and acting on data information [8]. Internet of Things (IoT) is recognised as one of the main technological mediums that allow sharing information between systems and subsystems within a smart building [4,25]. In addition, IoT has opened up an opportunity for connected objects that can improve serving customers' individual needs [26] and collect information to drive the development of more interconnected systems and subsystems [27]. Another study by Ref. [28] focused on the use of IoT to provide an interdisciplinary-based approach towards energy efficiency for buildings. whilst studies on IoT value for buildings have significantly advanced, focuses are often impacted by technology capabilities and the limited focus on how it can improve experiences of building users. A recent study by Ref. [29] pointed out that the use of IoT within buildings tend to generate large amount of data, which in many cases result in potential complexities in managing the data, data protection and optimisation. More importantly, in the context of buildings, IoT implementation is impacted by the nature of operations that vary based on type of building, which imposes another level of complexity when connecting IoT to different building users’ needs and expectations. The next section discusses Blockchain technology as one of the latest technologies that emerged within the construction industry.

In the context of data management and security, blockchain technology can be recognised as one rapidly developing technology that allows the safe recording of information while securely ensuring that information is protected against changing or hacking [9]. Blockchain is seen as a self-sustaining network that allows the creation and distribution of new blocks across nodes on the network [30], facilitating the transfer of digital assets among users without any intermediaries [31]. At a building level, there are many on-going efforts that elaborated on the value of blockchain technology with relation to different construction technologies. At a BIM level, studies that integrated the use of blockchain have mostly focused on the security related aspects. For instance, a study by Ref. [32] highlighted that value of blockchain for collaborative BIM platforms and distributed environments against potential threats such as confidentiality, integrity, authenticity, and availability. Another study by Ref. [33] discussed the implications of using blockchain to provide more secure collaborative design in BIM projects. The study pointed out the value of blockchain in improving security of design information through preventing data manipulation or denial of accessibility. Similarly, in the context of construction projects, many studies have shown promising outcomes of deploying the use of blockchain [34,35]. At an IoT level, as pointed out in the previous section, IoT systems generate massive volumes of data, which requires processing, storing and transforming it into meaningful information. Accordingly, blockchain provide means to record the transactions of data generated in IoT systems [36], and provide more verifiable, secure and robust mechanism to store and manage the data.

In the context of smart buildings, a study by Ref. [36] discussed the impact of BIM, IoT and Blockchain as digital construction technologies in the constitution of smart buildings. The study indicated that the synergy between these digital construction technologies can support more efficient management of data and information related to building components. Many later studies (e.g. Refs. [10,37] recognised that there is an effective synergy between these BIM, IoT and Blockchain as digital construction technologies to provide value across different aspects in buildings, and in many cases, can be linked to smart city development. However, evidence of the synergy between these digital construction technologies and smart city development to satisfy building users remain vague, hence this research will focus on this aspect. The next section highlights the interconnections between digital construction technologies in smart buildings and smart city.

### 2.2 Digital construction technologies in smart buildings and smart city

A city can be recognised as a complex system that is constituted of different systems such as transport, healthcare, energy, education, and environment, and these systems should work together and provide real-time information for each other when required [38]. For a smart city, these city systems should perform efficiently, effectively, and be able to generate accessible and useable information for other systems [39]. Seamless communication amongst these systems to make a ‘whole’ city through city-systems integration is a necessity to bring all city components together [3]. In addition, the availability of information from all city systems is a crucial requirement for a city to become smart [7]. Hence, transforming data generated by all city systems (e.g. transportation data, energy consumption data, data related to buildings) as well as social media and other devices to useful, accessible, efficient, and shareable information provides an environment in which city-systems access their required information from any context in real-time [40].

Amongst different constituents of smart city, smart buildings can be recognised as a key component contributing towards many smart city sectors including energy, water, infrastructure and sustainability [41]. Smart Buildings are equipped and supported by automated systems such as IoT with a primary aim to monitor the environment within a smart building and retain a storage of valuable information [5]. Whilst IoT is seen as one of the main facilitating technologies for smart buildings, it is also considered as a key infrastructure in a smart city as it has become one of the most used types of infrastructures [38]. The application of the IoT in the smart city comprises the capabilities of various devices, from smartphones and cars to refrigerators, that can be connected and share information, especially the ones within various city systems. It is recognised that IoT is at the core of smart city efforts and is the enabling technology that connected smart buildings’ data and information to smart cities [42]. As another supporting digital construction technology, and a key data infrastructure for IoT in smart buildings, the implementation of BIM for smart cities has been recognised as value adding through providing semantically rich information of buildings, which can benefit smart cities. For instance Ref. [43], proposed a holistic assessment of urban energy performance for smart city planning using BIM-GIS integration. Another study illustrated the use of BIM data at an urban scale in Singapore [44]. Another recent study by Ref. [45] proposed an inclusive framework integrating BIM-GIS to plan forecast utility infrastructure. In smart cities, the application of blockchain technology has been investigated across different aspects such as smart healthcare, smart transportation, smart grid, financial systems and supply chain management. For instance, in smart healthcare, blockchain technology was seen as an ideal solution to provide the desired level of decentralisation in healthcare networks, which effectively enhances security [46]. This is because the healthcare network comprises a group of hospitals, which are centrally controlled and this can subject the network to a single point of failure, hence decentralisation in healthcare environments is essential. In addition to decentralisation, blockchain facilitates storing, recording and sharing of medical data securely [47]. In smart transportation, blockchain can improve information sharing, support vehicle communication, can effectively handle the security and privacy issues related to the Intelligent Transportation Systems (Sharma et al., 2018). Blockchain was used for energy trading between distributed networks and electric vehicles to enhance the transparency of the system [48,49]. In supply chain management, blockchain has been proposed as an effective solution to track detailed product information and disallow the entry of forged products into the market [50]. A study by Sharma et al. (2019) proposed a blockchain-based solution to provide personalised and integrated services for the automotive industry. Another study proposed a blockchain-based information management scheme to address the poor traceability and fragmentation issues in the precast supply chain [49].

Amongst different digital construction technologies, it is recognised that IoT and Blockchain are the main interconnecting technologies between smart buildings and smart cities. This can be attributed to the fact that both technologies can be used at a building scale and also at an urban scale. Whilst research continues to exemplify the value of BIM within smart buildings, recognising value from smart city developers, compared to IoT and Blockchain, has considerably been limited [51] and this is the mainly reasoned by the inability to recognise the long-term value of the integrated data/information within the BIM process in terms of benefiting smart city systems [52]. Similar studies (e.g. Refs. [53,54]) also highlighted that limited evidence on how BIM data can support long term organisational development and value adding can impact the rationale behind BIM implementation. Many recent studies (e.g. Refs. [55,56] revealed data is fundamental to the usefulness and application, but more importantly, aligning how this data is coordinated, and used to improve buildings. Building users’ expectations in smart buildings can therefore be used as a mapping mechanism to understand the synergy between BIM, IoT and Blockchain as digital construction technologies to improve and utilise data in smart buildings, and also inform smart city developers by recognising value of data generated by smart buildings.

## 3 Research methodology

**Fig. 1:**
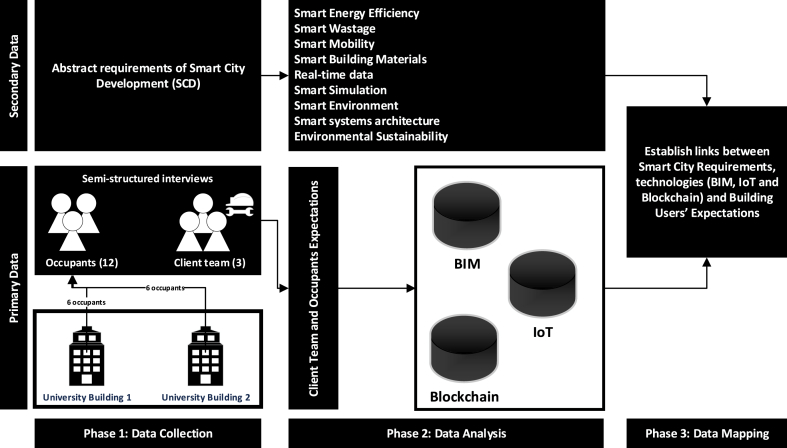
Proposed Research Design for the paper.

This research aims to draw a robust linking mechanism between digital construction technologies and Smart City Development to satisfy building users’ expectations. To achieve this, a qualitative-based approach using secondary from the literature review and primary data using semi-structured interviews. Fig. 1 provides an overview of the research design, which comprises of three phases. Table 2 is also provided to show the profile of research participants explaining their role in the building.

**Table 1:** Research participants’ profiles, roles, codes used in the analysis and relevance to the study.

Participant	Specific role (Code used in the analysis)	Relevance to the study
Client Team	Facility Manager (CT1)	Their perception plays a vital role in discussing issues about the building's in use face.
	Estates' Manager (CT2)	Their perception is vital to gain understanding on the strategies within university buildings as a whole.
	Information Manager (CT3)	Their perception is important to understand the information that they deal with, and what role it plays in management of the buildings.
Building Occupants	Staff Members (SM 1–4)	Their perception will support gaining better understanding of what impacts their daily jobs within the building.
	Students (ST 1–8)	Their perception will support understanding their experiences when using the university buildings.

**Table 2:** Questions asked during the interviews with both the client team and building occupants.

Question	Client Team	Building Occupants
What is your role?	Role	Use
From your experience, what are the complexities that you face within the building?	Management of the buildings	Use of the buildings (Included follow up questions)
As a building user, what are the expectations that you anticipate within the building?	Management of the buildings (Included follow up questions)	Experiences in the buildings (Included follow up questions)
In your opinion, how can operation and maintenance be improved within the building?	Issue management (Included follow up questions)	Issue reporting and experiences (Included follow up questions)
In your opinion, how can space use be improved within the building? How to address your expectations?	Space planning (Included follow up questions)	Space usage and experiences (Included follow up questions)
In your opinion, how do you think your technologies such as BIM can support you in improving the buildings?	Management of the buildings (Included follow up questions)	N/A

### 3.1 Data collection

The initial step taken was to derive, using secondary data from the literature, the requirements of Smart City Development (SCD) from a construction perspective. The significance of this step is that it will act as the base to map how building users' expectations can be addressed at Smart City levels. The authors therefore highlighted the key studies where construction and in particular BIM was linked to a smart city sector (see Table 3). The rationale behind identifying the requirements for smart city development from construction/BIM perspective is that BIM can be identified as the initial phase was data on buildings are inputted. In doing so, this will support identifying the link between BIM, other digital construction technologies including IoT and Blockchain and how they contribute to smart city development (SCD). As for the primary data, semi-structured interviews was used as the method to gather data from building users. Interviews allow richer understanding and holistic view into perceptions of building users, which is essential for the scope of this research study. In order to meaningfully and coherently respond to the research question, this paper uses a case study approach to contextualise the findings [57], and support a clearer response toward the complex phenomenon, which is the case in this research. It is noteworthy that the integration of an extensive literature review with the gathering of primary data through semi-structured interviews establishes a powerful mixed-methods approach, offering distinct advantages over relying solely on qualitative or quantitative methodologies. This unified strategy ensures a thorough understanding of the research problem by combining the depth of insights obtained from real-world experiences during interviews with the breadth of knowledge drawn from the literature [58]. This method allows for the exploration of contextual nuances, cross-verification, and triangulation of findings, enhancing the study's credibility and robustness. Additionally, this combined approach facilitates the development and testing of theories, providing practical and actionable insights. The inherent flexibility in this mixed-methods design allows researchers to adapt their focus based on emerging insights and ensures stakeholder involvement, with a specific emphasis on directly capturing user perspectives through interviews to foster a more user-centric understanding of the phenomena under investigation [59].

**Table 3:** Requirements of SCD from construction/BIM perspectives.

Requirements	Description concerning SCD	Authors
Smart energy efficiently	The use of real-time analysis to identify energy consumption patterns will provide energy savings as a result of more accurate energy monitoring. Efficient management of the resource in buildings, to deal with an energy shortage, such as the use of renewable sources of energy, allowing cities towards sustainability;	[16,43,51,60]
Smart wastage	BIM plays a crucial role in minimising wastage during construction utilising eco-friendly materials and recycling waste. Accurate quantity in BIM models identifies major waste management strategies and virtual and computational allows design before generating waste on-site.	[[61], [62], [63]]
Smart Mobility	BIM and GIS together can help smooth traffic management and green mobility in the cities. For example, the use of new technologies (e.g. wireless power transmission for charging electric vehicles) in the buildings, considered at the BIM implementation level help in several important requirements of SCD.	[63]
Smart Building Materials	To achieve sustainability by ensuring materials used in construction are, eco-friendly, secondary use, re-cycling and, utilization, purchase and installation price, transportation, produced from local resources	[60,63]
Real-time data, data sharing and exchange	Integration of building information generated in the planning and construction phase with operation phase through BIM: hence, providing dynamic and real-time information for SCD purposes. Collect, analyse and interpret real-time data and share it with interested parties.	[16,64]
Smart simulation	Building information modelling to simulate, risk management, environmental, quality, safety and progress prediction and control.	[7]
Smart Environment	Observe and analyse, and adapt to the external environment and the ability to receive information from vested parties.	[65,66]
	Use of historical information to plan and manage resources.	
Smart systems architecture	Technological components of a smart city. These components should be connected and integrated to provide services that are needed for a smart city. In addition, existing information generation, management, analysis tools, and techniques within buildings (e.g. BIM and GIS) should be integrated to provide dynamic analysis of both horizontal and vertical data to be utilised for SCD.	[1,67]
Environmental sustainability	Using “Green” technologies for construction and building facilities helps deduct harmful emissions for the environment so that moving towards environmental sustainability, is required by SCD. In addition, various green building standards, rating systems, and compulsory certifications that are recently required by some municipalities are great steps for making the construction industry, through the implementation of BIM, an important enabler of SCD.	[60]

For this research, one of the UK University buildings were chosen as they were delivered using BIM. The rationale behind focusing on two buildings is that one represents a generic university building that can be used by multiple faculties whereas the other includes specialist spaces that can primarily serve practical work (e.g. art, radio and media) led studies. It is anticipated that a focus on such two different nature buildings would help to elaborate on some of the complexities related to the expectations of both the client team and building occupants. The targeted sample within the case study were two categories: client team and building occupants. For clarification, a ‘client team’ in this context refers to an individual who helped manage and operate the building with respect to the business agenda and proposition for the building owners/organisations' sake. Meanwhile, an ‘occupant’ refers to either a student or visitor within the building. In total, 15 face-to-face semi-structured interviews (see Table 1) were conducted where 3 participants represented the client team and the other 12 were building occupants from the chosen university buildings. For the purpose of this research, reliability of the data collected did not rely on achieving saturation point, and this can be attributed to scope of this research. According to Ref. [68], in qualitative research, number of interconnected factors including research scope, quality of the data, and analytical approach impact the sample size. Within the context of this research, reliability of the findings did not rely on size of the sample interviewed, but more on the quality of data collected and analytical approach taken. In achieving appropriate quality, all interviews were transcribed and communicated with the participants. In addition, where possible, participants had follow-up questions during the interview to understand their experiences in the building, as this will support a more informed analytical approach when linking expectations to different digital construction technologies. The client team within the university is referred to as the “estates department”, hence the number of interviewees was pre-determined, as the client team that looks after the buildings primarily is led by the three interviewed participants. As for building occupants, a random sampling of staff and students were selected where respectfully six participants (2 staff and 4 students) were recruited from each of the chosen buildings. It can be stated, for this research, the number of interviewed occupants was not a concern for two reasons. The first reason is that the research aimed to explore what can be proposed to link BIM to smart cities, hence detailed approach toward specific data/information was not necessary. The second reason is that ‘expectations’ in their nature include a high level of subjectivity, and cannot be fully encompassed.

For the client team, the scope of questions was around the complexities they face in managing different buildings, expectations, usefulness of BIM-based data, and the potential of advanced digital construction technologies in improving the buildings. As for building occupants, the questions focused on their experiences within the building, complex issues faced and anticipated expectations. Despite the difference between questions for both the client team and building occupant, they served the same purpose, as they were worded differently to best suit the stakeholders. Such questions allowed the researchers to in-depth understand the stakeholders’ expectations. The data collected from the semi-structured interviews were analysed and processed via manual coding to derive themes for thematic analysis and discussion to translate the raw data into a format, which can be better understood and interpreted. To provide better clarity on questions asked for the participants, Table 2 is provided.

## 4 Research findings (data analysis)

This section presents the findings derived from both: the secondary data using literature, and primary data using semi-structured interviews. Through an in-depth analysis of data, this section firstly offers the findings of literature analysis regarding the requirements of SCD from the construction industry and BIM. Secondly, the findings from semi-structured interviews will be represented using thematic analysis.

### 4.1 SCD requirements from the construction/BIM perspectives

Based on the analysis of the literature, 3 highlights the main requirements of SCD from a construction point of view. As represented in the table, several key dimensions of a smart city have construction-related elements, which can be addressed by careful consideration of SCD through the implementation of IoT-enabled BIM.

### 4.2 Thematic analysis

**Fig. 2:**
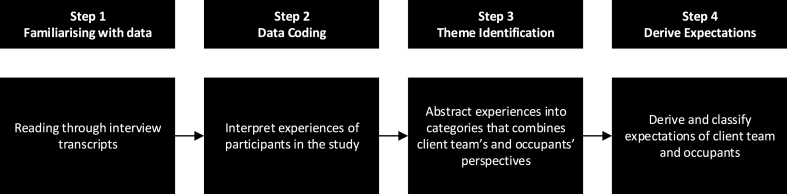
Process followed to analyse the primary data.

The transcribed raw data from research participants have initially coded into broader codes: complexities in buildings, anticipated expectations in a building, and application of technologies. To avoid potentially long quotes, selected responses were included in the below findings. For the purpose of this research, thematic analysis was used for the qualitative data collected using interviews. The approach toward identifying the themes is based on [68], which abstractly uses line-by-line coding. To illustrate the process of primary data analysis, the below figure (Fig. 2) is provided.

In order to illustrate an example of the above process (see Fig. 2), the below table (Table 4) is provided to show an example of the coding process followed to derive the analysis. This will help to identify how themes were derived, and provide clearer narrative for the discussion.

**Table 4:** Example of the coding process followed to analyse the data.

Data coding	Theme identification	Expectation	Quotation Examples
Issues faced by the client teams	Complexities in buildings	Coordination	Client team: “*More controls over lighting and heating can be helpful*”
Occupants' view of technology to improve their experiences in buildings.	Application of technologies	Navigation Space planning	Building occupants: “*It depends on the data which they take from me … so like I mean, if it*'*s without my approval then I would definitely disagree, that*'*s totally wrong, to be honest. As long as I know which data is being taken then it's all fine*”

#### 4.2.1 Complexities in buildings

Throughout the interviews, both the client team and building occupants were probed about their expectations for a building. Interestingly, while distinct differences surfaced between the client team and occupants, no significant variations were identified among occupants within the two chosen buildings. Initially, all participants were asked to define what they considered crucial for an effective and efficient building. The client team predominantly highlighted the importance of asset performance within the building, placing less emphasis on internal/external design and the occupants' needs. Notably, two participants from the client team extensively discussed how assets like Heating Ventilation Air Conditioning (HVAC), electrical systems, and Information Technology (IT) facilities contribute to optimising building efficiency. “*The assets that run the building to us are like the running oil, so it needs continual health check, monitoring and regular maintenance so that it does not breakdown. But in many cases, as proactive as your try to be, in many instances it will fall short”.*

Contrary to this, one of the participants from the client team has pointed out the importance of occupant experience in making a building successful, as it was stated.

“I think it’s mostly down to what the users get back out of it … so you can, you can design and build a stunning building but if it doesn’t perform very well or that the users are not happy with it then it fails as a building, that’s my idea”.

In addition, the client team were questioned about the issues and complexities that they face when managing the buildings investigated. Results showed most occupants enjoyed the building design, layout, and availability of space, whilst there was also reference to the quality of static features such as furniture. Nevertheless, issues of poor electrical components were also prominent, including the inconvenient locations of sockets and plugs, as well as limited accessibility to computers and their inadequate speed.

*“The heating and lighting should be well regulated and to my needs, with the general navigation of the building being easy to follow …. There needs to be good accessibility to electrical components too … … … More controls over lighting and heating can be helpful”* (CT1)

*“I would say we are almost there, with constant changes to the way we handle things I think we can definitely achieve this in the future”* (CT3)

*“I think it tried to deliver it, there’s always room for improvements and it’s how we learn than from those mistakes that we have made in the past and moving forward”* (CT2)

Most occupants emphasized the importance of safety, cleanliness, convenience, and comfortability in enabling building success. The clear difference here is that occupants focus more on the output of building features/assets, as opposed to the actual building features/assets that the client team explicitly specified. Based on the responses received:

*“As a staff member, I spend a long time sitting in particular spaces, so my experience is impacted by temperature, facilities and even the level of noise”* (SM1)

*“Ventilation is certainly an issue that can impact my experience in a space, and when an issue occurs, it is difficult to report it”* (SM3)

*“As a learner, my experience in teaching spaces can vary, but I find things like availability of electricity plugs, comfortable temperature, and ventilation are the most important to help me focus”* (ST3)

Based on the perceptions presented by different participants, it can be stated that complexities in the investigated buildings are impacted by intended functions (e.g. functions in a space), and also, intended expectations (e.g. ease to do a job). It is also apparent that current complexities can be seen as obstacles that prevent effective improvements, and this is primarily being faced by the client team. The next theme will further focus on expectations from both the client team and building occupants.

#### 4.2.2 Anticipated expectations in a smart building

All participants were directly asked about their expectations, with the client team queried on their expectations in managing the building, while occupants were inquired about their general expectations from the building. The goal was to better understand occupants' expectations regarding the buildings they use, in contrast to the client team's explicit focus on their organization's goals and business purposes, referencing data from BIM (further elaborated in the next theme). As a result, differences were evident between the expectations of the client team and those of the occupants. The findings indicated that occupants primarily prioritized comfort, cleanliness, security, safety, and ease of navigation within the building as their expectations. These factors were identified as key elements influencing the overall building experience for users. For example, two interviewees specifically emphasized the importance of these aspects. *“Have never thought about this if I am being honest, but I can say that it should be comfortable and clean”* (ST8)

*“Its important that everything is clean and tidy without any signs of damage which if present should be hidden away, so like cracks and stuff, also it needs to be safe and secure, and comfortable too of course”* (SM2)

*“In a University, I am mostly expected to use teaching space or working spaces, so when teaching, I find that temperature and ventilation have a high impact especially in long lectures, so it does not make me comfortable”* (SM1)

*“I know that many people use spaces, and I cannot expect everything to be perfect, but it is concerning when I see things that may impact my safety like open electricity boxes, or even when needing some privacy while working, it’s these things that affect my experience as a student”* (ST5)

Throughout all interview transcripts, the word ‘comfortable’ was mentioned most frequently, 32 times. Whilst ‘cleanliness’ and ‘layout’ were close by being mentioned on 17 and 11 different occasions. Moreover, it was noted the words ‘comfortable’, ‘comfortability’, and ‘comforting’ appeared in all occupants' requirements when questioned to order these critically, whilst 8 out of 12 occupants ranked these as either their first or second most important requirement. These are therefore the most fundamental occupant requirements which if met, can enhance performance quality. Generally, HVAC enables this, however greater control and adaptability of these features were expressed by few building occupants.

Assets and components are equally as important as they were also discussed. For instance, occupant 8 highlighted the importance of ‘accessibility’ and ‘navigation’ in prospect to the location of certain assets and features, in addition to the general flow of the internal building layout. This was also a common standpoint amongst many occupants. Such requirements fall under the concept of ‘space planning’, which is understood as a crucial requirement amongst nearly 50 % of all building occupants.

Client team on the other hand discussed other differing matters which frankly differed from occupant expectations, as evidenced from the below statements.

*“We need to seek decent standards or guidance documents which then everyone can refer back to ….as you know academics having their sayings and opinions. Everyone being all stakeholder which is involved with our University like subcontractors for instance”* (CT1)

*“Well there’s a lot of that we retrieved from the BIM Models, but there is further data which we need in order to you know to plan for the future and make improvements to what we currently have. But what we also need is full participation from everyone if I’m being honest. It just allows us to make better-informed decisions and progress by avoiding any disagreements or conflicts. We want the buildings to operate at their best too”* (CT3)

*“The future scope is very important to everything we do, so all our operations are done in that consideration …. With that being said we need to always have a good understanding of where we are as a business and educational institution. A key consideration to our institutions' aims and objectives are vital … …..occupants come first followed by the aims and objectives”* (CT2)

From the above responses, it can be stated that client team were not concerned about their experience within a building, but instead more focused on the business strategy, future scope, coordination, participation, and collaboration. It was also important to note that BIM, although used to execute both buildings, it was not pointed out as a significant support for effectively managing the building. The information manager in that regard pointed out that in many cases data derived from the BIM models cannot be directed towards the organisational’ s strategy or solve many of the complex issues faced within the buildings. As for occupants, and reflecting on their experiences, they stated the below:

In summary, based on the occupants' perceptions, it was clear that space planning, cleanliness, comfortability, security, safety and navigation were key occupant expectations, whilst business strategy, future scope, coordination, participation, and collaboration via data were the client team's expectations. From this, it can be argued that client team are interested in measurable objective data to achieve their requirements, whilst occupant data is more so subjective. The gap between client team and occupant expectations can perhaps be bridged to deliver greater end-user value via BIM. However, the previous discussion revealed BIM was inefficiently being utilised by the organisation.

#### 4.2.3 Application of digital construction technologies

From a data and technology viewpoint, the interview process also revealed significant similarities to how the client team and occupants perceived the use of data and technology for improved outcomes. It was clear that such stakeholders acknowledged and understood the potential of data for greater effectiveness and efficiency from their expectations, perspectives, and needs. However, this was based on certain conditions, which entailed the protection of their privacy, approval of data use and extraction, and avoidance of data misuse. For example, one interviewee said:

*“It would be a benefit definitely, it depends on if that’s the way we're going to go then will just have to rewrite all the security to ensure that you know the way we are moving forward is visible and people can say that’s what I signed up to, so then you know taking everything and then saying you this is what we are capturing, so as long as it is transparent with what we are capturing I don’t think it will be a problem, but of course, there will always be some people who have an issue with it”* (CT2)

*“Technology is great, and as most of our buildings were BIM-based, we expected that data retrieved will help decision-making and long-term improvement, but this was not the case. We still have to re-collect data and re-store them in BIM Models, which are not linked to the data we retrieve in the BMS system we have, so really it is challenging to judge the usefulness of such technologies to us”* (CT1)

From a client team's perspective, there was a strong commitment and belief that technologies such as IoT and even blockchain, which supported Industry 4.0 and SCD, could in essence deliver greater benefits. Despite viewing data as a valuable tool, extant data from BIM was seen as limited almost by all participants from the client team. This suggests data must be used for its best-fit purpose rather than irregularly. Furthermore, it was found that client team also discussed the need for transparency in explicitly stating their intended use of data, this directly falls in line with General Data Protection Regulations (GDPR), meanwhile addressing most occupants' concerns. For instance, one of the occupants stated the following:

*“It depends on the data which they take from me … so like I mean, if it’s without my approval then I would definitely disagree, that’s totally wrong, to be honest. As long as I know which data is being taken then it's all fine”* CT3)

It can be argued if data and technology are used to deliver better outcomes, it is vital to ensure people are informed about the intended use of their collected data without any misuse or misconduct. If this can be achieved, most of the occupants interviewed said they would be more than happy to allow for this, as supported by the following exemplar statement:

*“If this technology is used to benefit me in any way and that I know what the data is being used for then, in that case, I would be more than happy for the installation of this technology”* (CT2)

*“We always hear about the implementation of a smart technology or application, but never got to understand how it helps my experience in the building, as most of the existing problems remained the same without improvements”* (CT1)

Based on the above responses, it can be stated that the link between technology and its role to support different expectations is considerably lacking, and requires careful consideration to understand its role in building users. Although the university used BIM for delivery and implemented automated systems such as IoT throughout the two buildings, building users believe that more clarity should be sought to reflect how it can be used for improving and optimising the building.

#### 4.2.4 Summary of the findings from the thematic analysis

**Fig. 3:**
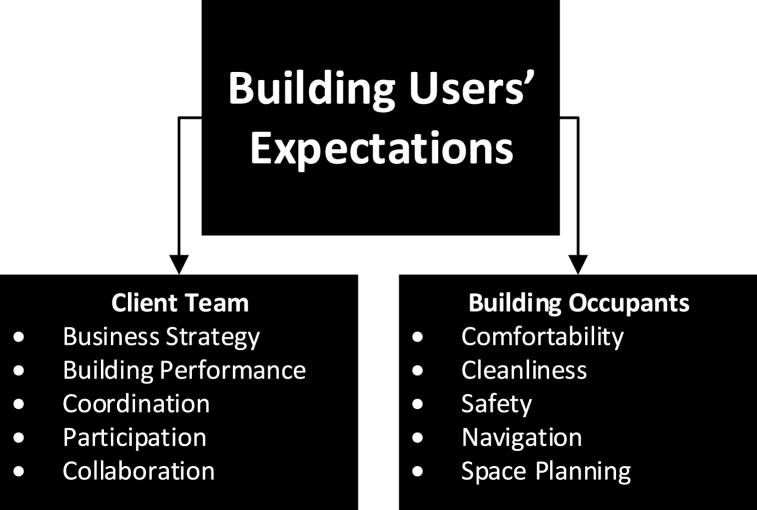
Key stakeholders' expectations based on the thematic analysis.

Therefore, based on the responses above, it can be stated that building users' expectations between the client team and building occupants are considerably different, and require careful consideration. More importantly, with the complexities faced by both the client team and building occupants, it can be realised that some of these complexities can be managed in the short term, but many others, especially for occupants, require continual monitoring and improvement, hence can be classified as long-term expectations. On the one hand, short term in this instance, refer to daily operations that support the primary functions of the building, so this applies when the building does not undergo any major changes (e.g. change of use of spaces, change of scope of the organisation). It is important to stress that this mainly applies to the client team, as they have the main decision about the business strategy and future scope of the building. Long term, on the other hand, refers to the continual optimisation, which happens over long period of time (e.g. yearly or following many years) to improve building performance, responding to occupants' needs and improving occupants' experiences. Fig. 3 shows the summarised expectations from both the client team and building occupant perspectives. Hence, the discussion will focus on mapping different digital construction technologies and Smart City to support satisfying different building users’ expectations.

## 5 Discussion (data mapping)

Examining the analysis we discussed earlier, it's evident that addressing the diverse needs of people involved in building projects presents challenges on two fronts. First, aligning advanced construction technologies with the preferences of building users proves challenging. The data from project managers and building occupants differed; managers sought concrete data and figures to meet their objectives, while occupants prioritized what felt right for them. Despite these differing needs, it's important to consider both perspectives for overall improvement. Second, linking all the smart solutions planned for an entire city to each individual building poses difficulties. Consider elements like smart waste management—tailoring these solutions for everyone in a specific building can be complex. While many studies have attempted to connect building data with Smart City requirements, the benefits for building users often lack specificity and need adjustment based on the building type (e.g., a university or a store). Therefore, our discussion aims to explore how we can integrate advanced construction technologies with Smart City plans to better meet the expectations of building users.

### 5.1 Bridging expectations of building users

The analysis showed that the nature of expectations by client team and building occupants within the targeted case study have differed. Based on the client team's perception, their expectations can be positioned within short and long-term expectations. As a short-term expectation, coordination and collaboration heavily rely on data/information available to manage daily operations [69,70]. In that respect, BIM can be used as a common data environment to obtain or retrieve data [54] about the building, which includes 3D visualisations, real-time information and documentation [71]. However, with the continual efforts in research and practice to adopt and implement BIM, integrating client team as part of the execution process remains a challenging issue [72],and this often results in incomplete or inaccurate data/information about the building during the in-use phase [73]. This consequently can result in poor coordination of operation and maintenance of building systems, and also poor management of spaces [11,35,74], which, based on the analysis, illustrate many of the client team's expectations in the short term. This indeed can reason some of the shortfalls that automated systems such as IoT may lack in terms of improving building performance on the long term. Aside from the complexity of IoT systems itself [75], the data collected by IoT systems is often recorded on a Building Management System (BMS), and in many cases, do not support the client team's decision making on different aspects within the building. Intrusively, the lack of data availability or accuracy for the short-term expectations can perhaps influence client team's long-term expectations.

**Fig. 4:**
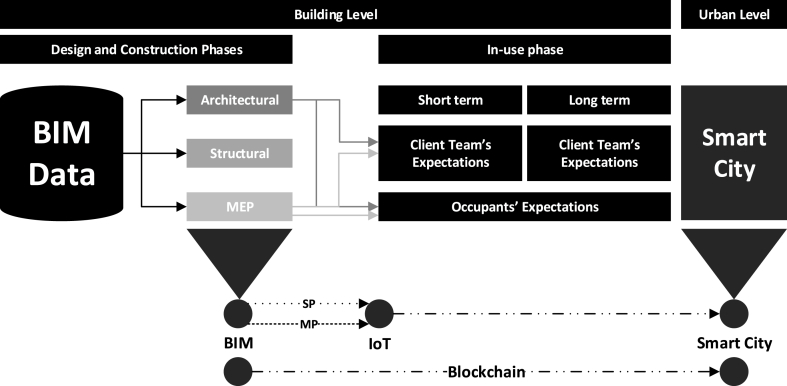
Positioning of Building users' Expectations with respect to digital construction technologies and Smart Cities.

In the context of this study, on the one hand, and based on the primary analysis, the client team outlined that data retrieved from BIM is often not integrated within smart systems such as IoT, and also highlighted that there are security implications that need to be considered. To illustrate this, Fig. 3 shows the process of data (Architectural, Structural, Mechanical, Electrical or Plumbing ‘MEP’) journey starting from BIM. BIM data that is retrieved can be 2D, 3D, schedules, cost and building life cycle data [76]. The lack of integrating BIM data within smart systems such as IoT, in addition to technological complexities, can be attributed to the limited participation of the client team in the BIM process which results in limiting the scope of BIM data when deploying the use of smart systems such as IoT. Scope of BIM data, in this context refers to the purpose of the data, which can be single purposed (SP) or multi-purposed (MP). In this instance, SP data refers to data that is produced for a particular purpose, whereas MP data refers to data that can inform multiple purposes. For instance, a study by Ref. [45], investigated the use of BIM data to plan utility infrastructure, which perhaps demonstrates an SP use of data. On the other hand, if some of the data used to plan utility infrastructure can potentially be used to plan maintenance, then this data becomes an MP set of data. It can be stated that lack of understanding scope of BIM data can result in poor synergies between different digital construction technologies, and can limit their role in satisfying building users' expectations. Although many studies have highlighted the issue of poor integration of building users' expectations as part of building delivery (e.g. Ref. [77]), this study supported highlighting a more complex issue, which is understanding scope of data. Lack of understanding scope of BIM data can impact the effectivity of automated systems such as IoT, and more importantly, recognising security and privacy considerations using technologies such as Blockchain. Based on the analysis, it can be stated that many of the client team's and occupants' expectations rely on data derived from the architectural and MEP BIM data. For instance, MEP Data can be used to optimise building performance, improve coordination, supporting business strategy and even improving comfortability [78]. As for architectural data, it can be used to support navigation, space planning [79,80], control cleanliness [69] and measure safety [81]. Consequently, understanding the above expectations, capability of different digital construction technologies and the scope of data can create an improved workflow (see Fig. 4) so that these expectations can be achieved.

On the other hand, at a smart city development level, and referring back to Table 3, each sector as part of SCD aims toward satisfying the different requirements that mostly feed into users' expectations whether at a building (e.g. energy) or urban level (e.g. transportation). This in return requires large amounts of data that is captured on a real-time basis using automated systems such as IoT. Whilst there are vast number of studies on IoT within the context of Smart Cities, the focus is often on the technological aspect, and in many cases, cascading the benefits from a smart city level to building levels is complex, and in many cases, cannot support optimising different aspects to support building users' expectations. IoT outputs, according to existing studies, anticipate improving customers' needs [26,82], and perhaps optimise a building's use to deliver an improved performance [27]. Although there are tangible evidences (e.g. Refs. [46,47]) of the synergy between smart city, and other digital construction technologies such as IoT and Blockchain, the emphasis on cascading many of resulting benefits of these technologies in satisfying building users' expectations remain complex, and in many cases, difficult to align in a connected and logical workflow.

Therefore, based on the above, it can be realised that satisfying building users' expectations would require holistic consideration of data at both BIM and Smart City levels. On the one hand, at a smart city level, there is an over reliance on capturing large amounts of data in real-time, which tend to provide value at an urban level, but not necessarily building levels. On the other hand, at a building level, BIM data, when created, is aimed at supporting the building lifecycle, whether at the design and construction level (e.g. for designers, contractors and sub-contractors) and at operation and management (for building users' including client team and occupants). Thus, it becomes logical to perceive that satisfying building users’ expectations would require improved understanding of workflows. The next section proposes a linking mechanism that supports a more tangible recognition of this connection.

### 5.2 Digital construction technologies in smart buildings and smart cities: a linking mechanism to satisfy building users’ expectations

The previous section highlighted the need to provide a more focused approach on scope of data between digital construction technologies and smart city development to satisfy building users’ expectations. Concerning BIM, it is acknowledged that many recent studies have focused on taking an end-point (Top-down) based approach, to understand different data requirements. For instance, one of the latest studies (e.g. Refs. [49,83], which focused on BIM implementation in handover management for an underground rail transit project, suggested that an operations-focused handover process is more practical to understand different data requirements within the BIM process. This was recognised as a cultivated mechanism to understand the value of asset-related digital data, and how their use can be deployed following the handover of a BIM project. It is important to note that simultaneous efforts continue to recognise the significance of focusing on data (e.g. Refs. [[84], [85], [86]]) within BIM projects. However, albeit these studies are potentially exposing the value of data within BIM, the level of complexity increases when considering the progressive use of data at an automated systems level, such as IoT, or advancing its use to be considered at the smart city level. This can perhaps be reasoned by the over-ruling of technology that aggregates data to produce outputs at the IoT or smart city level. Within a smart city, the data that feed into different city sectors (e.g. Energy, Transportation, etc.), when connecting to buildings, can perhaps be originated at a BIM level, before it commences to IoT output, and later becomes smart city-data. This connecting chain becomes tangible when understanding scope of the data at a BIM level, is a Single Purposed (SP) or Multi-Purposed (MP).

**Fig. 5:**
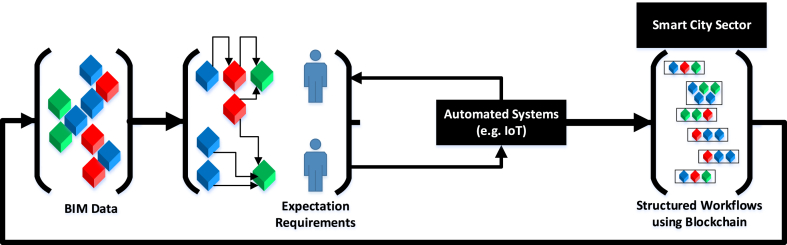
Proposed Linking Mechanism between Digital Construction Technologies and Smart City to satisfy building users' expectations.

It can be stated that satisfying building users' expectations require defining scope of BIM data forms a core element and this demands effective integration of different parties including the client team, and potential building occupants. Fig. 5 illustrates the role of digital construction technologies and smart city development in providing more informed facilitation of building users' expectations. According to Fig. 5, understanding expectation requirements is a key to link different data sets (represented as green, blue and red cubes) originated at a BIM level, and communicated to, according to the context of the study, the building users who are represented by the client team and building occupants. The understanding of expectations can elicit the role of BIM data (single or multi purposed), and therefore can solidify the role of BIM data when integrated with automated systems such as IoT. To support more coordinated approach towards BIM data, blockchain technology can be recognised as an effective, trusting and traceable technology [87]. In using blockchain technology, within the context of this study, it will support decentralising workflows [88], which will support a more tangible recognition of building users' expectations. Referring back to Fig. 5, the key role that blockchain plays is providing a more structured approach towards workflows, which provide simultaneous values. The main value is providing a more traceable approach that lines building users' expectations with BIM data where this can support more informed outcomes when using automated systems such as IoT. In this case, Blockchain acts as an effective mechanism in decentralising workflows [89] allowing more recognisable outcomes for different stakeholders. Another value is that, using the mechanism of structured workflows at a Smart City level so that it can inform BIM Data at a building level. This indeed can support informing the supply chain within construction projects providing more traceable and connected information management approach [49]. This in return can support better cascading of Smart City value from an urban level to a building level, allowing improved accountancy towards building users’ expectations.

#### 5.2.1 Digital construction technologies and smart environment: example case

As one of the smart city requirements, a smart environment can be recognised as one of the major requirements that are integrated across many sectors including education, healthcare, economy, housing, government and office [65,66]. In alignment with the description provided of the smart environment in Table 3, the main aim is to observe, analyse and adapt to the external environment and the ability to receive information from vested parties.

**Fig. 6:**
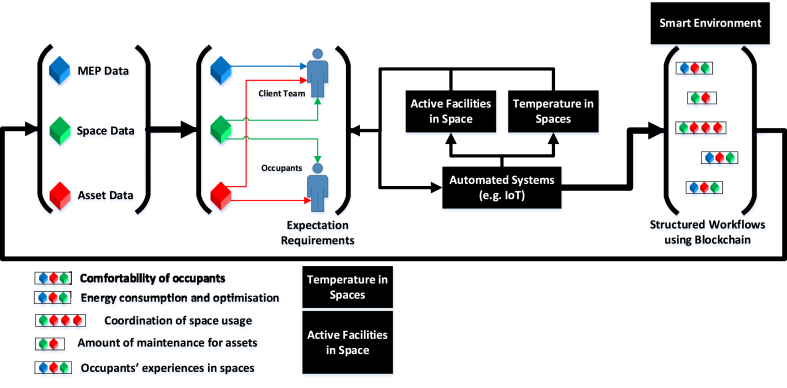
The synergy between digital construction technologies and smart environment as one of the sectors in SCD and how they can support satisfying building users' expectations.

Referring to Fig. 6, using BIM models, many datasets can be obtained from the architectural, structural and MEP models including specific parameters (e.g. thermal properties, material type, etc.) within these models. In this instance, MEP data, space data and asset data will be used to demonstrate the importance of defining scope of BIM data, how they link with different digital construction technologies and smart environment as one of the Smart City sectors. MEP data refers to data on the mechanical, electrical and plumbing systems where in many buildings, these systems are being monitored using building management systems. Space data refers to data on different spaces, including space type, functionality, accessibility and other attributes that define spaces in a building. Asset data refers to facilities that support the function(s) of a space which can include data related to type of the facility, health and safety, operation and maintenance. With reference to Fig. 6, it can be shown that space data and asset data are multi-purposed data that can support both the client team and occupants. In this respect, combining the MEP data, space data and asset data, IoT can support conveying temperature in spaces. The first example is on temperature in spaces, which can be linked to client team's expectation in terms of providing data on energy consumption and potential optimisation whereas for the occupants' it can obtaining information on comfortability in different spaces. The second example is, using the same set of data, IoT can support identifying active facilities in space where this can also support both the client team's and occupants' expectations. For the client team, it can support inform amount of maintenance and better decision making about space usage whereas for the occupants, it can support understanding experiences in spaces. In these two examples, blockchain technology can be used to provide structured workflows through recording the combination of different sets of data. These structured workflows can support informing smart environment as one of the smart city sectors. In return, over time, multiple structured workflows collected from other buildings at a smart city level with particular emphasis on smart environment, can support informing existing BIM datasets, which can play a key role in responding to building users' expectations.

Establishing a robust linking mechanism between Digital Construction Technologies and Smart City initiatives emerges as a crucial effort to effectively cater to the varied expectations of building users. This mechanism acts as a sophisticated interface, seamlessly integrating data from cutting-edge technologies such as Building Information Modelling (BIM), Internet of Things (IoT), and blockchain. Operating in real-time, it orchestrates the convergence of essential data types, including environmental metrics and occupancy patterns, facilitating dynamic adaptation to the evolving needs and preferences of building users. The comprehensive discussion underscores the necessity of identifying these critical data types, employing strategic application methodologies, and taking into account diverse stakeholder perspectives, encompassing building users, client teams, and Smart City developers. Real-world case studies and examples vividly illustrate the tangible application and benefits of the linking mechanism. Furthermore, the discourse emphasizes the paramount importance of scalability and adaptability, ensuring the mechanism's effectiveness across varied urban landscapes. In addressing potential integration challenges, the discussion provides solutions for issues like technological disparities, data silos, and resistance to change. The section concludes by pointing towards promising future research avenues, envisioning continuous refinement and evolution of the Linking Mechanism. In essence, this comprehensive framework not only bridges the gap between Digital Construction Technologies and Smart City initiatives but also offers a nuanced perspective for shaping an enhanced and user-centric building experience.

## 6 Conclusion

The paper explored the synergies between digital construction technologies with Smart City Development to satisfy building users' expectations. Literature review showed that there are extensive efforts on the application of digital construction technologies including BIM, IoT and Blockchain, and many studies have looked into how such technologies interlink with Smart Cities, yet limited studies have focused on the synergies between them in satisfying building users' expectations. For the nature of the study, and to gain holistically meaningful insight into the investigated phenomenon in this study, a qualitative-based inquiry using secondary data from literature and primary data using semi-structured interviews with building users (client team and building occupants) was used. Secondary data analysis showed that SCD from Construction/BIM perspective is often abstract and does not incorporate building users' expectations tangibly. Primary data analysis pointed out that building users’ expectations have differed, and satisfying these expectations, for the client team, can be positioned in the short and long term, but for building occupants, would mostly require long term due to the need of continuous improvement on building performance.

Based on the analysis derived, it was highlighted that the complexity in meeting building users' expectation lies two levels: mapping digital construction technologies to meet building users' expectations, and at a smart city level in terms of the complexity of cascading all smart solutions from an urban to a building level. Defining scope of BIM data was suggested as an effective mechanism to support an improved mapping between digital construction technologies to meet building users' expectations. At a smart city level, the use of structured workflows can support better recognition of value towards building users. The research proposed a linking mechanism between digital construction technologies and smart city development to satisfy building users’ expectations. Future research can explore the impact of the proposed mechanism in improving some contemporary technologies including digital twins so that a wider impact of data can be achieved. The paper acknowledges that one of the key challenges is how to efficiently handle data transitions between digital construction technologies and smart city development.

Employing a mixed-methods strategy that encompassed an extensive literature review and primary data collection through semi-structured interviews, this study acknowledges the pivotal role of qualitative insights from building users (client team and occupants) in shaping conceptual aspects, particularly the presented schemas. The qualitative nature of the data derived from these user interactions played a crucial role in formulating the proposed linking mechanism between digital construction technologies and smart city development. Furthermore, the research unfolded within the confines of a university setting, facilitating a controlled exploration of digital construction technologies. However, this setting's specificity may limit the generalizability of findings to diverse contexts, such as office buildings, mixed-use structures, or public buildings. Additionally, the study refrained from evaluating the conceptual model in a real-world setting, presenting a potential limitation in establishing more robust connections between digital construction technologies and smart city development. Moreover, despite the extensive exploration of building users' perspectives, a limitation is evident due to the absence of direct input from smart city developers or stakeholders. The inclusion of their perspectives could have added depth to the research by providing insights into the extent of integration between smart city initiatives and smart buildings. Despite these acknowledged limitations, the study imparts valuable insights into the intricate relationship between digital construction technologies and smart city development. It underscores the importance of data scope in meeting building users' expectations and introduces a linking mechanism to enhance this integration.

Findings from this paper can inform existing body of knowledge in terms of stressing the importance of data scope in the context of different digital construction technologies, and how it links to different stakeholders. More importantly, the study exemplifies further potentials of how smart city development can be used to inform data on buildings, which can add to the growing knowledge areas in construction such as Artificial Intelligence, Machine Learning and potentially Metaverse. For industry, the study can inform future client teams or facility managers on elements that impact building performance. More importantly, the study stresses the importance of integrating building users as part of BIM-based projects, and in informing different expectations. Another implication from this study, is the added value of using blockchain as a way structure workflows, and trace data requirements where this add value of both achieving expectations, and cascading value streams from Smart City level to building levels.

## Data availability

Data included in article/supp. material/referenced in article.

## CRediT authorship contribution statement

**Mohammad Mayouf:** Writing – review & editing, Writing – original draft, Validation, Methodology, Investigation, Formal analysis, Conceptualization. **Fiaz Afsar:** Writing – original draft, Validation, Methodology, Conceptualization. **Ayzah Iqbal:** Writing – original draft, Methodology, Data curation, Conceptualization. **Vahid Javidroozi:** Writing – original draft, Validation, Methodology, Data curation, Conceptualization. **Saeed Reza Mohandes:** Writing – review & editing, Writing – original draft, Validation, Formal analysis.

## Declaration of competing interest

The authors declare that they have no known competing financial interests or personal relationships that could have appeared to influence the work reported in this paper.
